# Implementation Research to Inform the Use of Xpert MTB/RIF in Primary Health Care Facilities in High TB and HIV Settings in Resource Constrained Settings

**DOI:** 10.1371/journal.pone.0126376

**Published:** 2015-06-01

**Authors:** Monde Muyoyeta, Maureen Moyo, Nkatya Kasese, Mapopa Ndhlovu, Deborah Milimo, Winfridah Mwanza, Nathan Kapata, Albertus Schaap, Peter Godfrey Faussett, Helen Ayles

**Affiliations:** 1 ZAMBART Project, University of Zambia, School of Medicine, Lusaka, Zambia; 2 National TB program, Ministry of Health, Lusaka, Zambia; 3 Clinical research Department, Faculty of Infectious and Tropical Diseases, London School of Hygiene and Tropical Medicine, London, United Kingdom; Boston University, UNITED STATES

## Abstract

**Background:**

The current cost of Xpert MTB RIF (Xpert) consumables is such that algorithms are needed to select which patients to prioritise for testing with Xpert.

**Objective:**

To evaluate two algorithms for prioritisation of Xpert in primary health care settings in a high TB and HIV burden setting.

**Method:**

Consecutive, presumptive TB patients with a cough of any duration were offered either Xpert or Fluorescence microscopy (FM) test depending on their CXR score or HIV status. In one facility, sputa from patients with an abnormal CXR were tested with Xpert and those with a normal CXR were tested with FM (“CXR algorithm”). CXR was scored automatically using a Computer Aided Diagnosis (CAD) program. In the other facility, patients who were HIV positive were tested using Xpert and those who were HIV negative were tested with FM (“HIV algorithm”).

**Results:**

Of 9482 individuals pre-screened with CXR, Xpert detected TB in 2090/6568 (31.8%) with an abnormal CXR, and FM was AFB positive in 8/2455 (0.3%) with a normal CXR. Of 4444 pre-screened with HIV, Xpert detected TB in 508/2265 (22.4%) HIV positive and FM was AFB positive in 212/1920 (11.0%) in HIV negative individuals. The notification rate of new bacteriologically confirmed TB increased; from 366 to 620/ 100,000/yr and from 145 to 261/100,000/yr at the CXR and HIV algorithm sites respectively. The median time to starting TB treatment at the CXR site compared to the HIV algorithm site was; 1(IQR 1-3 days) and 3 (2-5 days) (p<0.0001) respectively.

**Conclusion:**

Use of Xpert in a resource-limited setting at primary care level in conjunction with pre-screening tests reduced the number of Xpert tests performed. The routine use of Xpert resulted in additional cases of confirmed TB patients starting treatment. However, there was no increase in absolute numbers of patients starting TB treatment. Same day diagnosis and treatment commencement was achieved for both bacteriologically confirmed and empirically diagnosed patients where Xpert was used in conjunction with CXR.

## Introduction

There has been an increase in the notification of smear negative pulmonary TB especially in high HIV prevalent settings.[1–4] This is largely due to the diagnostic challenge posed by HIV associated TB which includes pauci-bacillary disease resulting in a low sensitivity of sputum smear microscopy in this population.[3, 5–7]In contrast to the pre-HIV era, smear negative TB patients in high HIV prevalence settings have been observed to have poor outcomes and these may be as a result of delayed diagnosis or incorrect diagnosis of TB. [8–10]In recognition of these challenges, the World Health Organisation (WHO) has issued several guidelines aimed at improving the diagnosis of TB in high HIV prevalence settings.[2, 11–14]

In 2006 and 2007, the WHO issued guidelines for improving diagnosis of smear negative TB in high HIV prevalence settings and on use of liquid culture in low and middle income countries.[2, 13] These guidelines recommended early use of Chest X-ray (CXR) and use of culture in smear negative HIV positive presumptive TB patients.[1]Implementation of these guidelines faced ongoing challenges such as the limited access to culture and CXR. [15] Apart from high cost, need for specialised laboratories, and specialised training, the long turnaround time(TAT) to culture results limited the impact of culture in on of TB. CXR use was limited by non-availability at peripheral facilities as well as by the difficulty in interpreting results largely due to scarce qualified staff with capacity to correctly interpret CXRs.[16].In 2010, the WHO issued the first guidelines for use of Xpert MTB RIF (Xpert) in high burden TB and HIV settings in further recognition of the diagnostic challenge TB still posed in these settings.[12] Replacing culture with Xpert could result in shortening the TAT especially if placed at peripheral facilities.[17–19]The challenges of CXR availability and interpretation issues could be resolved through use of novel imaging techniques that might replace conventional radiography. The recent advances in digital technology with the use of computer aided diagnostic (CAD) systems may potentially overcome the challenges that have faced use of CXR in resource constrained settings.[16, 20–22]Digital CXR technology with automated scoring is now available. [23]

In view of the considerable cost of Xpert which limits the extended use of Xpert especially in resource constrained settings, guidelines for Xpert recommended use on pre-selected patients considered to be at high risk for TB and in whom use would most likely have the most impact.[11] The cost of using Xpert can also be minimised through use in conjunction with other screening tools that identify high risk patients such as CXR.[24, 25]The initial guidelines recommended Xpert as an initial diagnostic test for suspected HIV associated TB and for suspected MDR TB. Later, the guidelines were revised to include use of Xpert as a second line test in patients with smear negative results or those with an abnormal CXR without HIV infection or those with unknown status but not seriously ill as a conditional recommendation.[11] Apart from shortening the TAT for results availability to one day, the use of Xpert was also expected to increase TB case detection and improve outcomes of TB patients. There is mixed evidence available now showing that whilst use of Xpert increases accurately diagnosed TB, its impact on case detection may be undermined by continuing empirical treatment of TB. [18, 19, 26–28]

The WHO guidelines for use of Xpert were based on evidence from initial multicentre demonstration studies and were based largely on sensitivity and specificity data.[29, 30]Implementation of Xpert in resource constrained settings requires evidence to inform policy, especially when used in primary health care facilities. Further evidence is required on how to use Xpert within the recommended WHO guidelines.[11, 15]In this study, we conducted implementation research to inform the prioritisation of patients for testing with Xpert in a high TB HIV burden resource-constrained setting. Two algorithms were assessed for TB detection, using either HIV infection or CXR as entry points to accessing Xpert diagnosis.

### Ethics Statement

The study had ethical approval from the University of Zambia biomedical research ethics committee. The requirement to obtain individual written informed consent was waived for presumptive TB patients being screened as part of the case finding activities, as this was part of routine health care and all modalities of TB diagnosis included are currently recommended as best practice. Oral informed consent was obtained from presumptive TB patients at the time of registration to the presumptive TB register.

## Methods

### Study setting and study population

The study was conducted in two primary health care facilities in Lusaka, Zambia. The health facilities are located in peri—urban Lusaka and offer services to a population with a high burden of TB and HIV. In one facility, an algorithm using computerised scoring of digital chest radiography (CXR) as a pre- screening tool to select samples to test with Xpert was used. In the other facility, an algorithm using HIV testing as pre- screening tool was implemented. The facilities were selected based on their high burden of TB and HIV as well as based on the low proportion of confirmed cases starting TB treatment. The CXR algorithm site notified 2207 TB patients, with an overall notification rate of 1711 /100,000 population (based on an estimated population size of 129,000) in 2010. Of all notified patients, 1881 (85.2%) had pulmonary TB of which 533 (28.3%) were smear positive. On the other hand, the HIV algorithm site notified 831 patients, with an overall notification rate of 681/100,000 population (for an estimated population size of 122,000) in 2010. Patients notified with pulmonary TB were 568 (68.4%) and of these 179 (31.5%) had smear positive pulmonary TB. The HIV co infection rates among notified TB patients were over 68% in both facilities. According to the latest population estimates, in 2012, the CXR algorithm site had an estimated population of 161,000 whilst the HIV algorithm site had a population of 145,200 (personal communication sister’s in charge).

The study population included individuals that presented to the health facilities and met the definition of a presumptive TB patient. In this study, in line with the updated screening strategy proposed by WHO[11, 31] a presumptive TB patient was defined as any individual with a cough of any duration who was able to submit a sputum sample. Algorithm data presented was collected between March 2012 and March 2014 from consecutive patients presenting to the facilities during the study period. Data for comparison of notification included all notified TB register data for these 2 facilities from 2^nd^ quarter 2010 through to 1^st^ quarter 2014.

### Study Objectives

The main objective of the study was to evaluate 2 algorithms for prioritisation of patients for Xpert testing. The main outcomes of interest were: 1) to assess how pre-screening with CXR alone, CXR and HIV in combination and HIV alone would impact on the numbers needed to be tested with Xpert to detect TB; 2)to determine additional bacteriologically confirmed TB cases starting TB treatment; 3) to measure changes in notification rates after introduction of Xpert; 3) to assess the time to starting TB treatment for both bacteriologically confirmed and empirically treated patients and how this changed with availability of CXR;4) to determine the proportion with empirical TB treatment and 5) to determine the proportion of cases that are missed as a result of following the HIV algorithm.

### Study design and procedures

This was a prospective study. As part of the study, mobilisation activities were conducted in the community aimed at raising community awareness of tuberculosis and encouraging community members to present for TB screening. Mobilisation activities were conducted throughout the duration of the study. Details of the mobilisation activities have been described elsewhere.[32] Briefly, mobilisation activities were conducted through use of drama, mega phone announcement, distribution of flyers, health talks and door-to-door visits. Community members were encouraged to present to the health facility if they had a cough of any duration. Open access points were established at both facilities and these allowed patients to present directly to TB services without queuing up in the general outpatient clinic before being investigated for TB. A presumptive TB register was maintained at the open access point and all patients presenting for TB diagnosis were registered and allocated a unique presumptive TB patient number.

### CXR algorithm procedures

At the CXR algorithm study site, a digital CXR unit (Odelca-DR, Delft Imaging Systems, the Netherlands) located within the facility premises was used to perform CXRs of presumptive TB patients. Presumptive TB patients were offered a CXR, followed by HIV counselling and testing. The CXRs were automatically scored by a Computer Aided Diagnostic Program (CAD) developed to detect abnormalities suggestive of TB. (CAD4TB, version 1.08, Diagnostic Image Analysis Group, Nijmegen, the Netherlands) [23, 33]. The software was trained on digital X-rays obtained at two sites with a high TB prevalence in Sub-Saharan Africa. Details of the development of this software have been described elsewhere. [34–36] The CAD program scores the x-ray on an abnormality score ranging between 0 and 100, and a higher score is indicative of more severe abnormalities. [23] CXR data previously collected from the same population was used to select an appropriate threshold for the CAD score.[32] A threshold of 61 was chosen based on the area under the receiver operating curve (ROC). The ROC was constructed with a test set of CXRs collected in the same population using a radiological reference. We have previously described a proof of principle prospective study where we compared consecutive presumptive TB patients offered both Xpert and FM regardless of their CXR score to assess the sensitivity and specificity of the CAD program at the chosen thresholds. [32]. In this proof of principle study, the negative predictive value of CAD for TB detection was 100% and the positive predictive value was 33% using Xpert as a reference standard.

**Fig 1:**
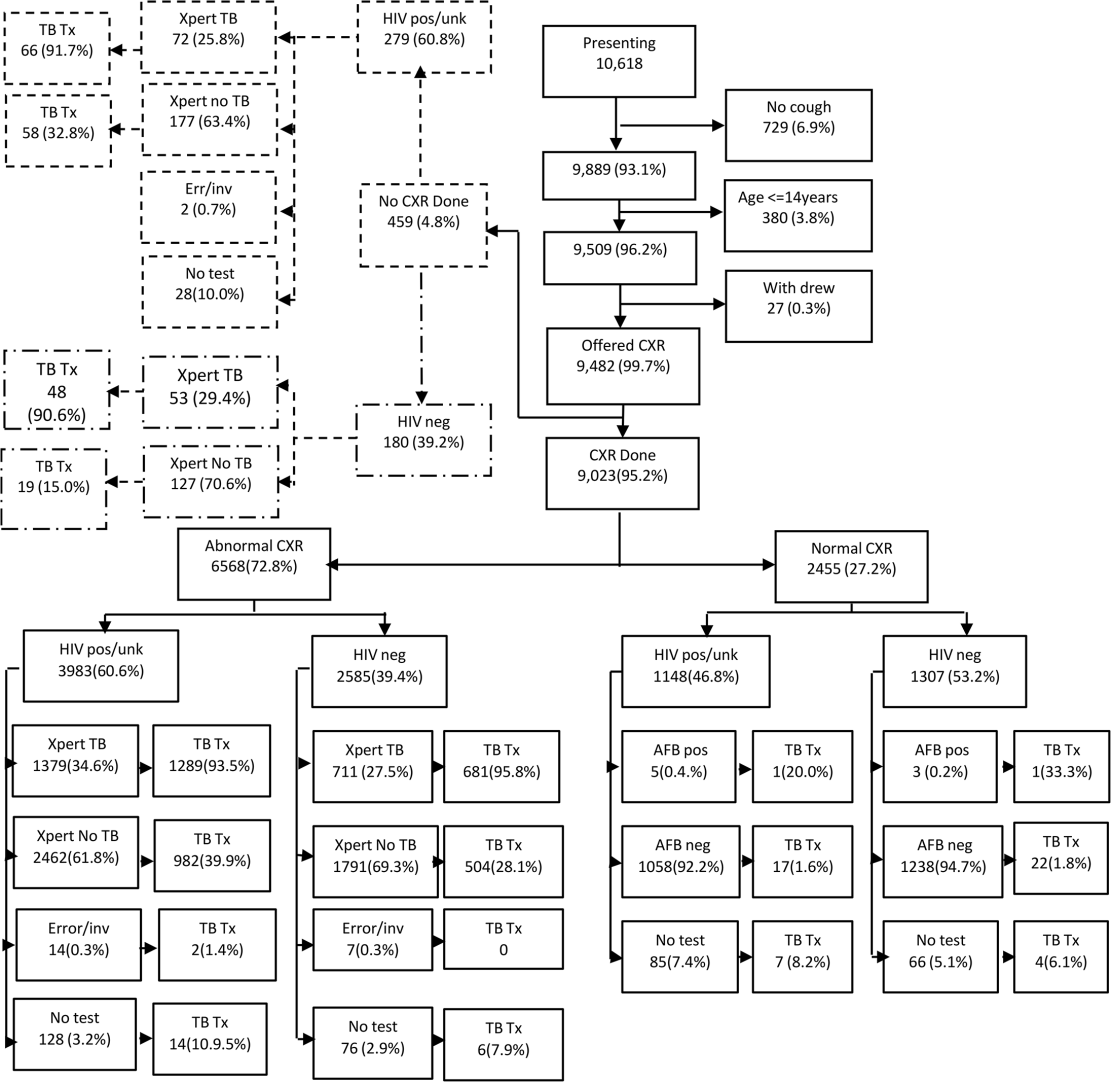
Flow of patients CXR algorithm.

For this study, using the same threshold of 61 as the cut off, a prospective study of consecutive presumptive TB patients presenting to the facility were enrolled over a period of 2 years. Patients were tested with Xpert if their CXR was scored as abnormal (CAD score 61–100) and FM testing was done if their CXR was normal (CAD score 0–60) regardless of their HIV status. Individuals that did not get a CXR done were tested with Xpert (Fig 1).

### HIV algorithm procedures

The HIV algorithm was designed in line with the WHO recommendations for use of Xpert in high TB HIV burden settings.[11, 12]In these settings, Xpert testing is strongly recommended as an initial diagnostic test in individuals suspected of HIV and TB co-infection. Working on this premise, presumptive TB patients presenting to this facility were offered counselling and opt out HIV testing, followed by Xpert test for those who were HIV positive and smear microscopy testing for those who were HIV negative. Additionally, HIV status unknown (those who declined to test for HIV) were also offered testing with Xpert.

In order to understand how many are missed by testing those who are HIV negative with FM, over a period of two months, consecutive presumptive TB patients were offered both Xpert and FM regardless of their HIV status.

### Sputum testing and TB diagnosis

Xpert MTB/ RIF (Cepheid, Inc., Sunnyvale, CA, USA) instruments and LED-FM microscopes were placed in both primary health care facility laboratories. Patients being tested with Xpert were requested to submit a spot sample, whilst those being tested with FM were requested to submit a spot and morning sample in line with the National TB Program guidelines. Xpert and FM testing were done according to standard operating procedures.[37, 38]Patients that did not have TB detected by either method were referred for clinical review and assessment, which included physical examination and review of CXR where available.

TB diagnosis was defined as bacteriologically confirmed TB where Xpert detected MTB or FM detected Acid Fast bacilli, or as clinical TB (empirical TB diagnosis) where TB treatment was commenced in a patient who did not have TB detected by either diagnostic method.

### Data capturing and analysis

All patients presenting to the open access points were registered and assigned a unique patient identifier number using an electronic data base. The database was designed and developed using Microsoft SQL Server 2005 (Microsoft Corporation, Redmond WA, USA) as the back end and Microsoft Visual Basic 2005 (Microsoft Corporation, Redmond WA) as the front end. Patient demographic details and history of symptoms data were collected during the registration process. Electronically generated CRFs were printed for each patient with the unique patient identifier number (barcode) and these were used to capture CXR, HIV, Xpert and FM testing data which was entered into the data base described above by scanning the printed unique identifier number. The database had five separate data entry screens to facilitate the data capturing of the different Case Record Forms (CRFs). The Patient Barcode was used as a unique identifier for all the tables and forms.

To facilitate linkage of the presumptive TB register data to the TB treatment register, a TB register data base was designed that captured all patients who were notified during the study period. Again the presumptive TB patient number was used as a unique identifier.

For analysis purposes, the data were linked to MS Access (Microsoft Corporation, Redmond WA, USA) via Open Database Connectivity (ODBC) and exported to STATA for statistical analysis (Stata Corporation Version 11. College Station, TX, USA). Initially a descriptive analysis was conducted to describe the population that participated in the study. A flow diagram was generated to show the flow of patients through the diagnostic process. To determine the time to starting TB treatment, the presumptive TB treatment register data were linked to the TB treatment register. The time to starting TB treatment was defined as the number of days between the date of first presentation to the diagnostic services and the date the patient was commenced on TB treatment. To determine the changes that occurred to the notification data, a before and after comparison was carried out to compare changes in absolute numbers of notified patients, and changes in proportions of pulmonary smear positive, pulmonary smear negative and extra-pulmonary TB as well as to determine the additional new bacteriologically confirmed TB cases commencing TB treatment after introduction of Xpert. The before period included notification data from 2^nd^ quarter 2010 to 1^st^ quarter 2012 whilst the after period was 2^nd^ quarter 2012 to 1^st^ quarter 2014. Additional TB cases were calculated by subtracting notifications for the corresponding period prior to the implementation period and the % change was calculated by dividing the additional cases found with the sum of notifications in the period before multiplied by 100. To demonstrate changes in proportions, pie charts were generated.

To determine if the observed changes in the proportion of bacteriologically confirmed cases after the intervention were attributable to the introduction of Xpert or to changes in the distribution of the TB suspect population, crude and adjusted proportions were calculated for new bacteriologically confirmed pulmonary cases before and after introduction of the intervention using new pulmonary TB cases as the denominator. Adjusted proportions were obtained using direct standardisation on age, sex and HIV status. The standard population was defined as all new pulmonary TB patients diagnosed during the before and after periods of the study for that community.

Further analysis was done to understand how the numbers required to be screened by Xpert would change if the CXR algorithm were used in combination with the HIV algorithm. As part of the CXR algorithm diagnostic procedures and as already explained above, all presumptive TB patients presenting to the CXR algorithm site were offered both CXR and HIV testing. Using the available HIV data and CXR data from this site, a simulated algorithm was generated that combined the CXR results with the HIV results. In this algorithm, the assumption made was that patients would be screened with HIV first, followed by CXR to discriminate patients into four groups of; 1) HIV positive with normal CXR; 2) HIV positive with abnormal CXR; 3) HIV negative patients with normal CXR and 4) HIV negative with abnormal CXR. A further assumption was made that only patients with abnormal CXR would have sputum tested whilst those with a normal CXR would not have any further testing since it has been demonstrated that the CAD program used in this study successfully identified patients who were list likely to have TB.[32]

Proportions were compared using the chi-squared test whilst the continuous variables time to starting TB treatment and age were compared using the T-test.

## Results

### CXR algorithm

#### Baseline results

At the study site where the CXR algorithm was tested, a total of 10,618 participants presented, of which 9889 (93.1%) met the definition of a presumptive TB patient of cough of any duration. After excluding children aged 14 and less (380) and those who withdrew, 9482 (95.9%) presumptive TB patients were included in the final analysis. Of these presumptive TB patients, 459 (4.8%) did not get a CXR done. The mean age of all study participants was 36 (SD 12.0) and 4925 (51.9%) were HIV positive. Of the 4925 HIV positive individuals, 3123 (63.4%) reported their HIV positive status as already known whilst 1802 (36.6%) were newly diagnosed. There were more males than females that presented for screening; 5853 (61.7%) compared to 3629 (38.3%) respectively (p<0.0001). The majority of patients, 6014 (63.4%) had a cough duration of 2 to 8 weeks. Among the 9023presumptive TB patients who had a CXR done, 6568(72.8%) had an abnormal CXR whilst 2455 (27.2%) had a normal CXR. (Fig 1 and Table 1)

**Table 1:** Baseline characteristics of study participants, CXR algorithm.

Total	9482(%)
**Gender**	
Female	3629 (38.3)
Male	5853 (61.7)
Mean age(SD	36(SD12.0)
**Chest X-ray**	
Abnormal CXR	6568 (69.3)
Normal CXR	2455 (25.9)
CXR not done	459 (4.8)
**HIV status**	
HIV negative	4072 (42.9)
HIV positive (already known)	3123 (32.9%)
HIV positive (Newly diagnosed)	1802 (19.0%)
Unknown HIV status	485 (5.2)
**Cough duration**	
Less than 2 weeks	2436 (25.7)
2–8 weeks	6014 (63.4)
> 8 weeks	943 (9.9)
Unknown duration	89 (0.9)
**Previous TB treatment**	
Yes	2126 (22.4)
No	7349 (77.5)
Unknown	7(0.1)

#### TB case detection and time to TB treatment

Of the 9482 presumptive TB patients, 9074 had evaluable results. A total of 2223/9076(24.5%) presumptive TB patients had TB detected by either Xpert or FM (Fig 1). Among those with abnormal CXR and valid Xpert result, Xpert detected TB in 2090/6343 (32.9%) whilst among those with CXR not done, Xpert detected TB in 125/429 (29.1%).In those participants with a normal CXR, FM detected TB in 8/2304 (0.3%). Of the 8 TB cases detected by FM, 4 CXRs were read as being consistent with TB by the radiographer whilst the CAD system scored them as normal, the rest were scored as normal by both the radiographer and by CAD. (Fig 1)

The median time to starting treatment for those with bacteriologically confirmed TB was 1 day (IQR 1–3) (Table 2).

**Table 2:** Median time to starting TB treatment.

Type of Patient	CXR Algorithm Median-days(IQR)	HIV algorithm Median(IQR)
All	1(1–3)	3 (2–6)
Bacteriologically confirmed GXP positive only FM positive only	1(1–3) 1(1–3) 4.5 (3–6)	3(1–5) 2(1–5) 3(2–5)
Bacteriologically unconfirmed GXP negative only FM negative only	1(1–3) 1(1–3) 7(3–75)	8(4–31) 12.5(5–33) 7(4–25)

#### Empirical TB treatment

Among presumptive TB patients with an abnormal CXR in whom TB was not detected by Xpert or did not have a sample tested, 1508/4478 (33.7%) were empirically treated for TB (Fig 1). Among those with a CXR scored as normal in whom TB was not detected by FM or did not get an FM test, 50/2447(2.0%) were empirically treated for TB. The median time to starting TB treatment for bacteriologically unconfirmed individuals with an abnormal CXR and tested with Xpert was 1 day (IQR 1–3 days) whilst for individuals with a normal CXR tested with FM, it was 7 days (IQR 3–75) (Table 2).

#### Impact on notification

**Fig 2:**
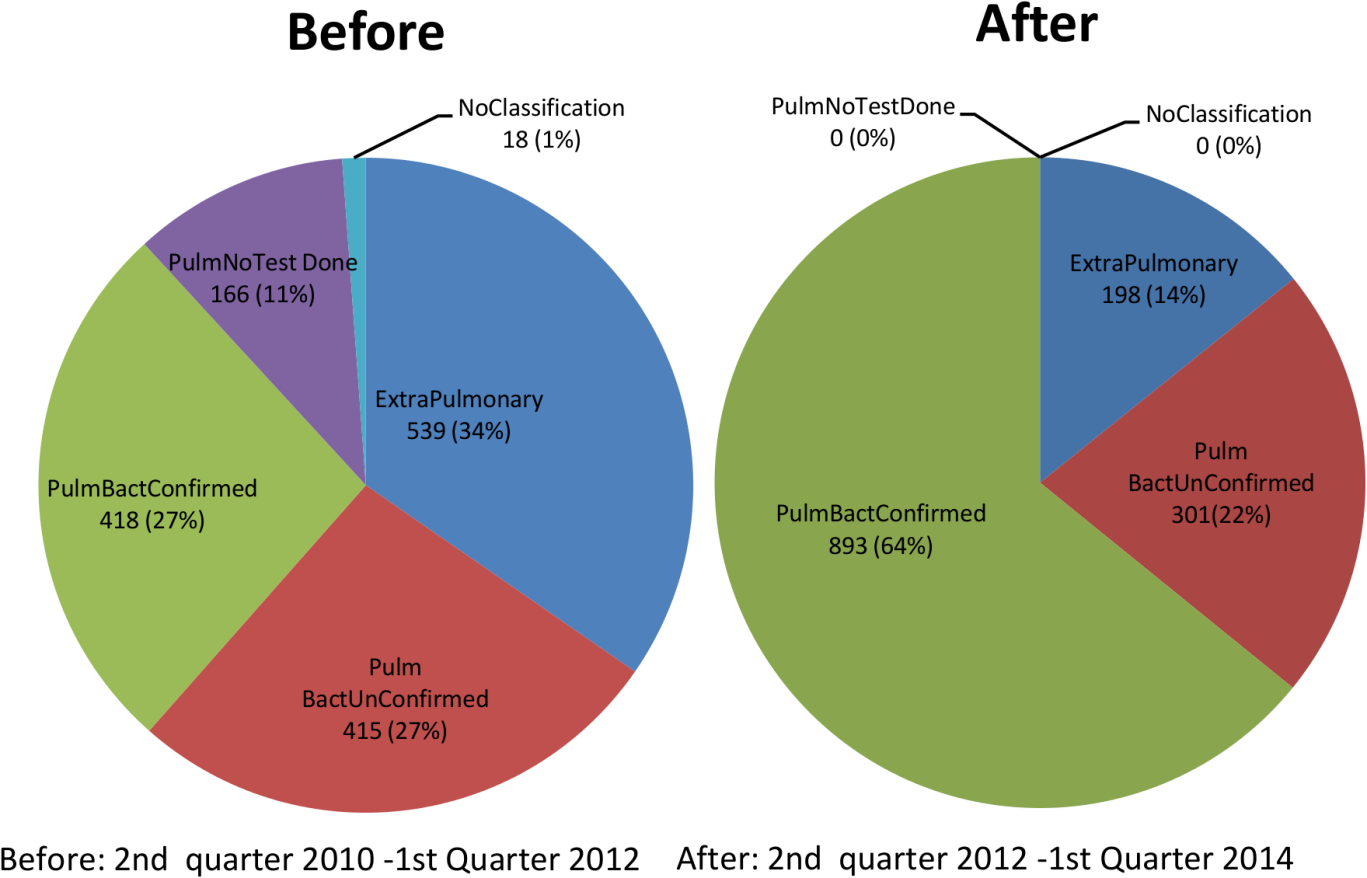
Comparison of before and after case notification at the CXR algorithm site.

Using notification data, 1045 additional new bacteriologically confirmed TB cases were notified during the intervention period compared to the period before the intervention translating to a percentage change 110.0% from baseline. Additional all forms of TB notified were 255 (6.0% change from baseline) (Fig 2). The notification rate for all forms of TB notified was 1652/100,000/yr population before the interventions and 1403 /100,000/yr population after the intervention using 2010 and 2012 population estimates respectively. The notification rate of new bacteriologically confirmed TB was 368/100,000/yr population before the intervention compared to 620/100,000/yr population after the intervention. (Fig 2A and Table 3) The crude proportion of new bacteriologically confirmed cases among new pulmonary TB cases before the intervention was 0.380 (95%CI 0.361–0.399) and after the intervention it was 0.666 (95%CI 0.649–0.683). The adjustment for age, sex and HIV status did not change the results significantly; 0.384 (95%CI 0.365–0.402) before the intervention and 0.667 (95%CI0.650–0.683) after the intervention.

**Table 3:** Crude and Adjusted proportions, new bacteriologically confirmed TB.

Community	Total	New Pulmonary TB Patients (bacteriologically confirmed)	Crude (95%CI)	Age/Sex/HIV adjusted (95%CI)
**CXR Algorithm Before After**	4263 4518	2499 (950) 2995 (1995)	0.380 (0.361–0.399) 0.666 (0.649–0.683)	0.384 (0.365–0.402) 0.667 (0.650–0.683)
**HIV Algorithm Before After**	1556 1392	696 (360) 976 (758)	0.517 (0.480–0.554) 0.776 (0.751–0.803)	0.515 (0.480–0.549) 0.779 (0.753–0.804)

### HIV algorithm

#### Baseline results

**Fig 3:**
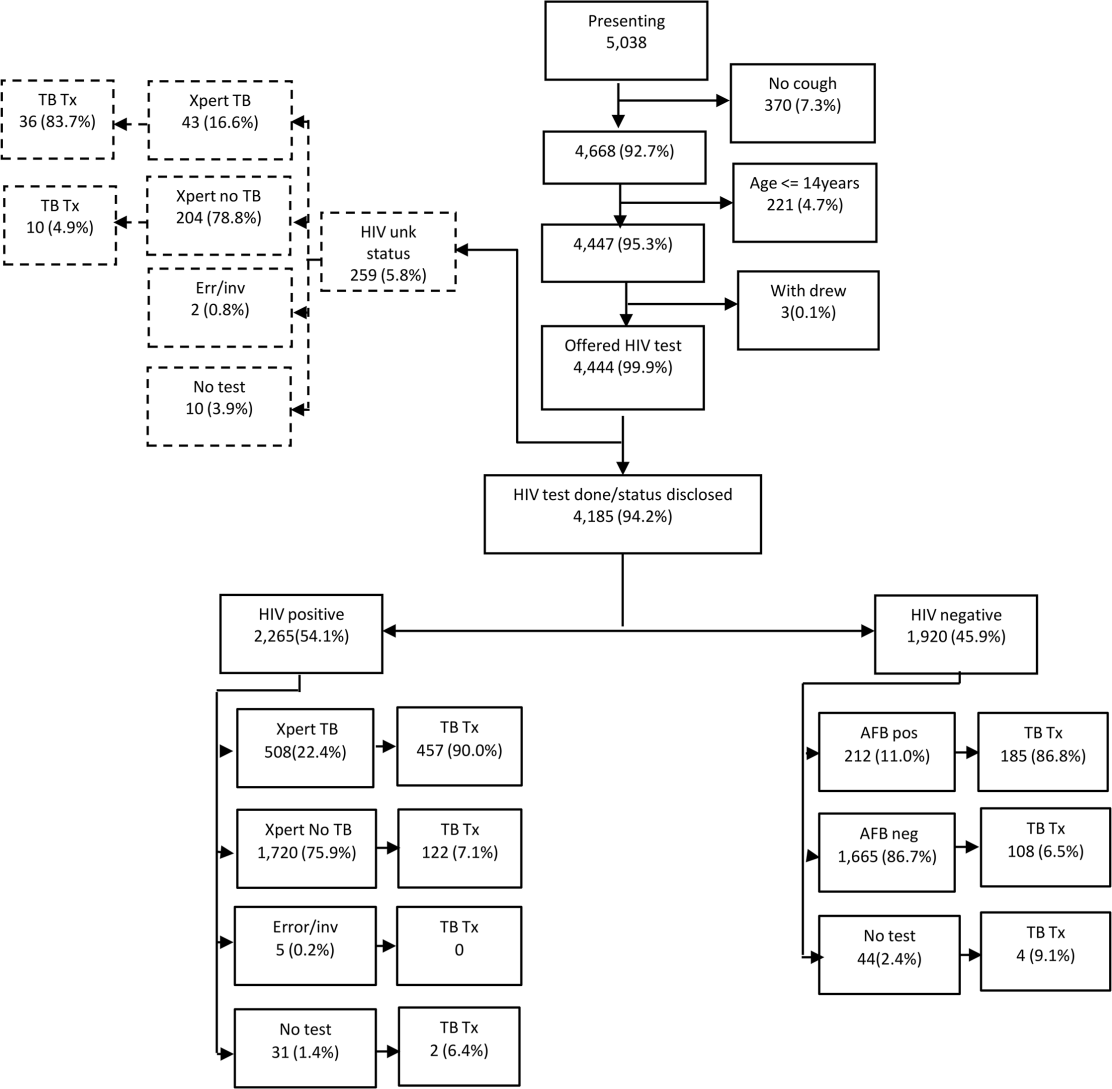
Flow of patients HIV algorithm.

At the study site where the HIV algorithm was tested, a total of 5038 individuals presented for screening of which 4668 (92.7%) met the definition of a presumptive TB patient (cough of any duration). After excluding children aged 14 years and less and those that withdrew, 4444 were included in the final analysis.(Fig 3)The mean age of all study participants was 36 (SD 12.0) and 2265 (51.0%) were HIV positive. Of the 2265 HIV positive individuals, 1620(71.5%) reported their HIV positive status as already known whilst 645 (28.5%) were newly diagnosed. There were more males than females that presented for screening; 2599 (58.5%) compared to 1845 (41.5%) (Fig 3 and Table 4)

**Table 4:** Baseline characteristics of study participants, HIV algorithm.

Total	4444(%)
**Gender**	
Female	1845 (41.5)
Male	2599 (58.5)
**Mean age(SD**	36.3(SD 12.1)
**HIV Status**	
HIV negative	1920 (43.2)
HIV positive (already known)	1620 (36.5%)
HIV Positive (Newly Diagnosed)	645 (14.5%)
Unknown HIV status	259 (5.8)
**Cough duration**	
Less than 2 weeks	1747 (39.4)
2–8 weeks	2286 (51.4)
> 8 weeks	351 (7.9)
Unknown duration	60 (1.3)
**Previous TB**	
Yes	872 (19.6)
No	3572 (80.4)

#### TB case detection and time to TB treatment

Of 4444 presumptive TB patients, 4352 had evaluable sputum results. A total of 763/4352 (17.5%) presumptive TB patients had TB detected by either Xpert or FM. (Fig 3) Among the 2265 participants who were HIV positive, Xpert detected 508 (22.8%) TB cases whilst among those 244 with unknown HIV status and valid Xpert results, TB was detected in 43 (17.4%). FM was AFB positive among 212/1876 (11.3%) who were HIV negative (Fig 3).

A total of 537 patients received both Xpert and FM tests regardless of their HIV status. Among these 238 (44.2%) were HIV negative of which FM was AFB positive in 22 (9.2%) whilst Xpert detected TB in 31 (13.0%). Among the 216 HIV negative individuals that were FM smear negative, Xpert detected TB in 13 (6.0%) individuals.

The overall median time to starting treatment for those with bacteriologically confirmed TB was 3 days (IQR 2–6). The median time to starting treatment for those detected using Xpert was 2 days (IQR 1–5) whilst for those detected using FM it was 3 days (IQR 2–5), (p = 0.072) (Table 2)

#### Empirical TB treatment

Among presumptive TB patients with positive or unknown HIV status in whom TB was not detected by Xpert or did not get an Xpert test done, 134/1972 (6.8%) were diagnosed with TB clinically and commenced on TB treatment. Among those with a negative HIV status in whom TB was not detected by FM,100/1709 (5.8%) were diagnosed with TB clinically and commenced on treatment. The overall median time to starting TB treatment for bacteriologically negative individuals was 8 days (IQR 4–31). When restricted to those with Xpert negative results, the median time to starting TB treatment was 12.5 days (IQR 5–32) whilst for those tested with FM it was 7 days (IQR 4–25) (p<0.0001)(Fig 3).

#### Impact on notification

**Fig 4:**
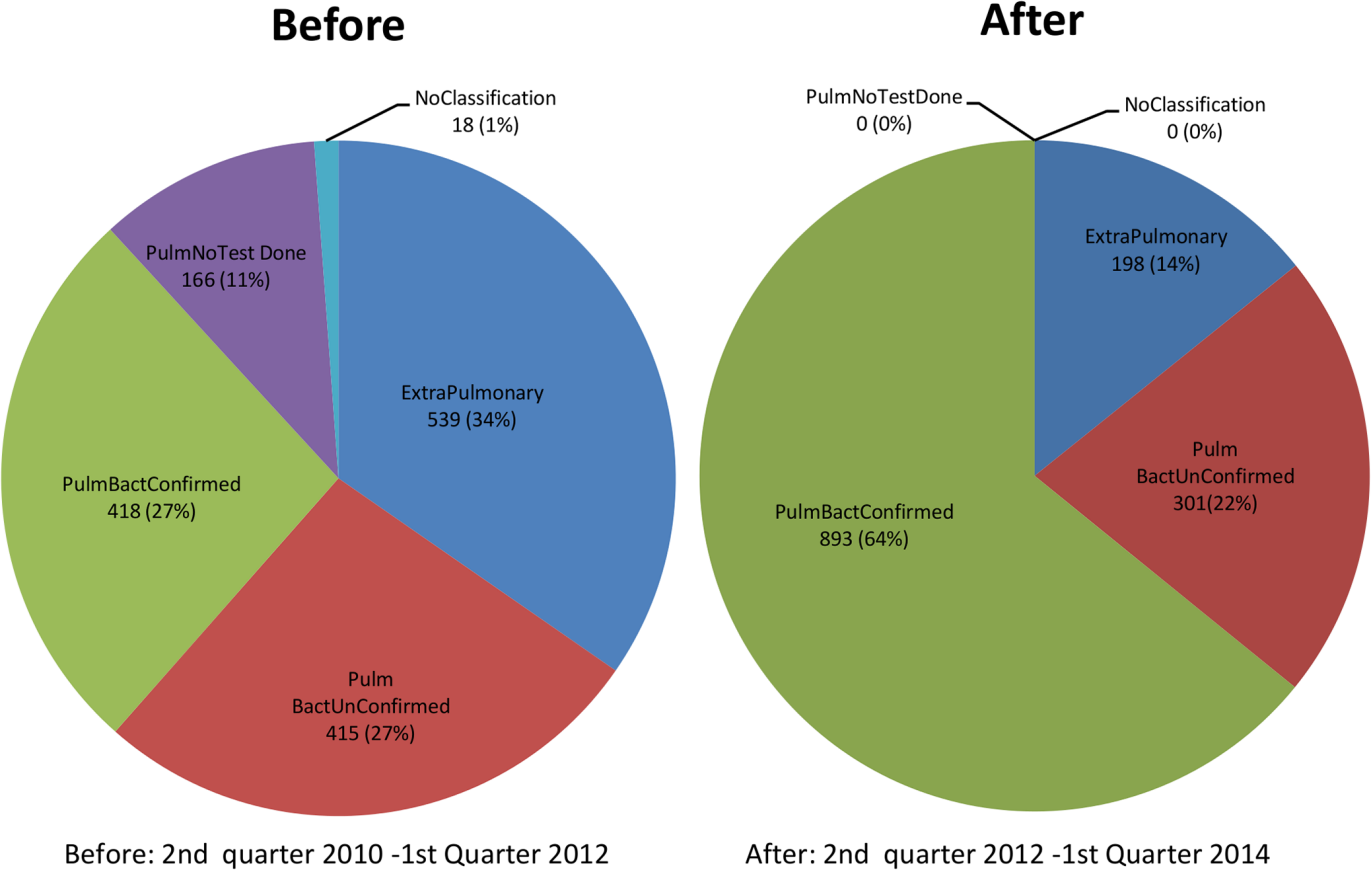
Comparison of before and after case notification at the HIV algorithm site.

Additional new bacteriologically confirmed TB cases notified were 398 representing a 111% change from baseline. There was a reduction in all forms of TB notified by 164 representing -11% change from baseline. The notification rate of new bacteriologically confirmed TB was 145/100,000/yr and 261/100,000/yr pre and post intervention respectively (Table 3). The notification rate for all forms of TB notified was 638/100,000/yr and 480/100,000/yr pre and post intervention respectively. (Fig 4, Table 3) The crude proportion of new bacteriologically confirmed cases among new pulmonary TB cases before the intervention was 0.517 (95%CI 0.480–0.554) and after the intervention it was 0.776 (95%CI 0.751–0.803). The adjustment for age, sex and HIV status did not change the results significantly; 0.515 (95%CI 0.480–0.549) before the intervention and 0.779 (95%CI 0.753–0.804) after the intervention.

### Simulated algorithm: combined CXR and HIV algorithm

**Fig 5:**
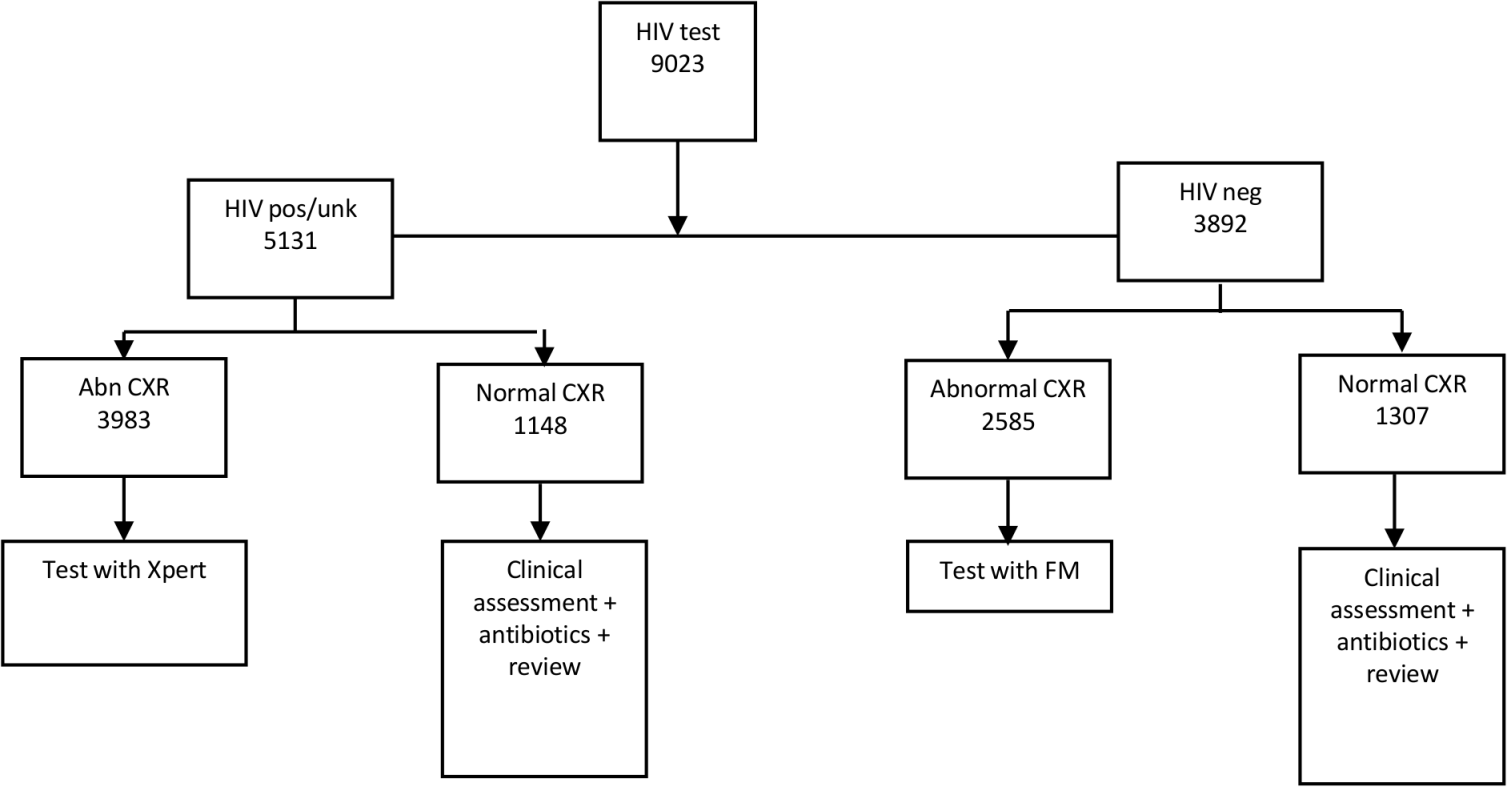
Simulated algorithm, combined HIV and CXR algorithm.

If the CXR and HIV algorithm are combined, out of the 9023 screened with HIV, 5131 (56.9%) were HIV positive or status unknown. Among these HIV positive individuals, if they all underwent CXR, 3983/5131 (77.6%) would have an abnormal CXR and these would be tested with Xpert. Similarly of the 3892 that were HIV negative, 2585 had an abnormal CXR and would be tested with FM. In this combined algorithm, 27.2% of those originally screened (2455/9023) would not have their sputum tested with either Xpert or FM whilst 44.1% (3983/9023) would be tested with Xpert and 28.7% (2585/9023) would be tested with FM (Fig 5).

## Discussion

The overall detection of TB using either algorithm was surprisingly high in this population where a widely inclusive definition of a presumptive TB patient was used; cough of any duration. This finding suggests that inadequate numbers of patients present for screening for TB and that there may be a pool of undiagnosed TB in the community even when a sensitive diagnostic tool, such as Xpert, is used in conjunction with case finding activities. This is also supported by the high number of abnormal CXRs among presumptive TB patients at the site where the CXR algorithm was implemented. These findings suggest that the use of better TB diagnostic tests will only have the desired impact if patients access diagnostic services in a timely manner and will require broader investment in community, health services to extend community case finding activities.

The CXR unit with computerised scoring used in this study resulted in 27% patients not being tested by Xpert. This is because the CAD program used to score CXRs was set to very high sensitivity and low specificity in order not to miss cases. This means that large numbers of patients still have to be tested with Xpert which may be a challenge for resource constrained settings. However, If used in combination with HIV pre-screening followed by Xpert testing only for those who are HIV positive with abnormal CXR, this would result in 56% patients not being tested with Xpert which potentially can reduce the cost of diagnosis. The marginal costs of digital CXR are small compared to the cost of cartridges. In a study to determine the sensitivity of the CAD program used in this study, done in the same population, it was found that individuals with a normal CXR score were unlikely to have TB, suggesting that no further screening is required for individuals with a normal CXR regardless of their HIV status but validation studies are still required using culture as a reference standard as opposed to using Xpert as was the case in this study.[32]In this combined algorithm, HIV negative individuals with an abnormal CXR could be tested with FM thus improving the sensitivity of FM and reducing the numbers needed to test. Doing FM test only in those individuals with abnormal CXR score could improve the sensitivity of FM and also potentially reduce the overall cost of diagnosis.

The CXR algorithm used in this study has shown that it is possible to use CXR and Xpert and still get a same day diagnosis and that placement of these diagnostic facilities at the point of use is vital for reducing time to treatment. In this study, the time to starting treatment in those with abnormal CXR and Xpert TB detected was one day and this is in contrast to observations from settings where Xpert is placed centrally.[17, 39, 40] Further, same day empiric TB diagnosis and treatment was also possible. It has been observed previously that empiric TB treatment initiation can take up to several weeks with a mean time to diagnosis of 13 days compared to 3 days for smear positive cases.[4, 41]The role of onsite placement of CXR in reducing time to treatment has been observed by other studies conducted in the same population.[19, 41]We also found that where CXR was not readily available (HIV algorithm site), a longer time to empiric TB diagnosis and treatment was observed with an overall median time to treatment of 8 days further highlighting the possible role availability of diagnostic tools at a point of care have in reducing time to diagnosis.

Using HIV screening to pre-select patients so that only those who HIV are positive or HIV status unknown accessed Xpert resulted in 43% patients not being tested with Xpert. If used among HIV negative patients as a follow on test to smear, Xpert detected TB in 6% of those who would have been missed by smear microscopy. The use of Xpert as a follow on test in smear negative TB patients who are not suspected to have HIV associated TB is a conditional recommendation by the WHO, but this poses ethical problems as well as resource problems. Smear negative patients who do not have HIV associated suspected TB should be considered for testing with Xpert as a follow on test to avoid delays in diagnosis and to prevent ongoing transmission from these patients. However the feasibility of such a strategy would need to be ascertained as it would require huge resource allocation to test large numbers of smear negative TB patients to find 1 case of TB among this population. Use of CXR as a further screening tool in smear negative HIV negative presumptive TB patients could reduce the number needing to be screened with Xpert as a follow on test.

At both study sites, the proportion of pulmonary smear negative (empirically diagnosed)TB expectedly reduced significantly after introduction of Xpert, however the overall number of cases notified remained the same or decreased slightly. This suggests that some cases that would have previously been diagnosed as smear negative were now diagnosed as bacteriologically confirmed cases and that there was likely to be reallocation of case status rather than increased numbers of cases detected. It is not immediately clear why the extra pulmonary TB diagnosis reduced drastically at the HIV algorithm site. The significant reduction in the number of extra-pulmonary TB diagnosis made after introduction of Xpert may explain the fall in absolute numbers of TB cases starting treatment in this facility.

Time to empiric TB diagnosis and treatment for patients examined using Xpert was 7 days longer than those examined by FM at the HIV algorithm site. These findings are contrary to the expectation that clinicians would more likely make a diagnosis of TB in patients suspected to have HIV associated TB especially in a high incidence setting. The proportion of patients with empiric TB treatment was the same in both HIV negative and HIV positive patients, again contrary to what would be expected.

The study has both limitations and strengths. The two sites were different, had different clinicians, were accessed by different communities and different algorithms were used and therefore cannot be directly compared. However, the strengths of this study are that it was conducted in high TB HIV setting area with Xpert placed at primary care sites within the routine health system. Data from these settings are scant and this study informs on how Xpert can be used in these settings.

## Conclusions

The routine use of Xpert in a resource-limited setting at primary care level resulted in additional cases of confirmed TB patients starting treatment expectedly and increased notification rates of bacteriologically confirmed TB cases. Same day diagnosis and treatment commencement was achieved for both bacteriologically confirmed and empirically diagnosed patients where Xpert was used in conjunction with CXR. The HIV algorithm in this setting reduced the number tested by Xpert by 43% whilst the CXR algorithm reduced the number tested by 27%. The CAD program used in this study was able to discriminate successfully between those with TB and those without TB. If used in combination with the HIV algorithm, the numbers tested with Xpert could be reduced by 56% whilst the numbers tested by FM could be reduced by 71%. The marginal costs of digital X-ray are small compared to the cost of Xpert cartridges. Using the recommended WHO algorithm alone results in missed cases among those who are HIV negative and therefore not tested by Xpert and use of Xpert as a second line test in these patients should be considered though the feasibility and cost benefit of this strategy would have to be ascertained in view of the huge resources that would be required to test large numbers of smear negative patients to find 1 case of TB. The problem of missed cases can be reduced by using CXR in combination with HIV testing to screen patients. Cost effectiveness of such strategies would have to be ascertained.

Finally the findings in this study inform on the use of CXR as a pre-screening tool for active TB as recommended by the WHO in their new recommendations for TB screening. [42] The use of computerised scoring of CXRs has the potential to extend the use of CXR in resource constrained settings which still face challenges of qualified staff to interpret CXR correctly and thus has the potential to minimise the cost of TB diagnosis especially when used in conjunction with expensive new diagnostic tools.

## Supporting Information

S1 Data Aggregated notification data. (XLSX)

S2 Data CXR algorithm data. (CSV)

S3 Data HIV algorithm data. (CSV)
