# Correction: Implementation Research to Inform the Use of Xpert MTB/RIF in Primary Health Care Facilities in High TB and HIV Settings in Resource Constrained Settings

**DOI:** 10.1371/journal.pone.0137934

**Published:** 2015-09-03

**Authors:** Monde Muyoyeta, Maureen Moyo, Nkatya Kasese, Mapopa Ndhlovu, Deborah Milimo, Winfridah Mwanza, Nathan Kapata, Albertus Schaap, Peter Godfrey Faussett, Helen Ayles

The incorrect image appears for Fig 2, “Comparison of before and after case notification at the CXR algorithm site”. Please view Fig 2 here.

**Fig 2:**
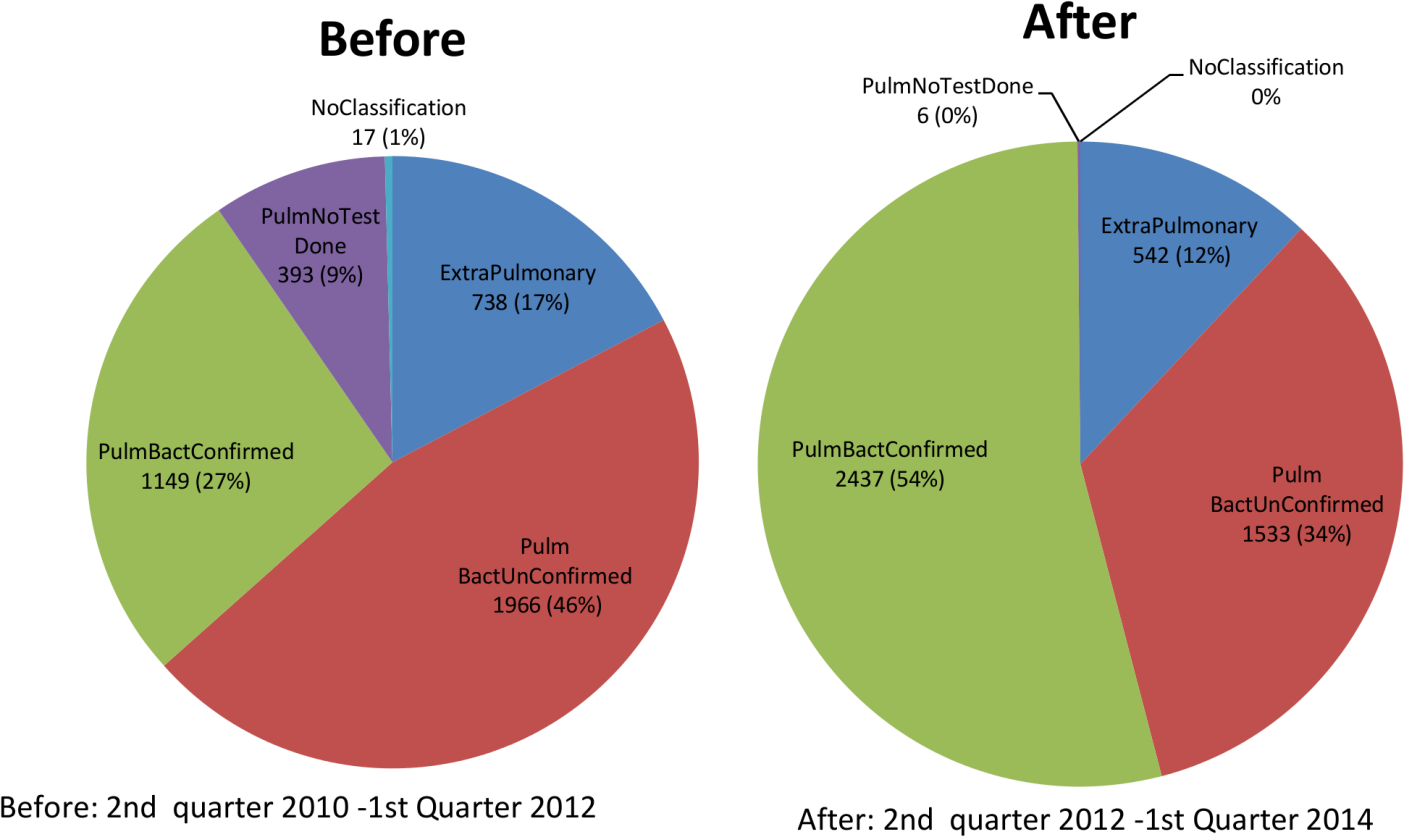
Comparison of before and after case notification at the CXR algorithm site.

The fourth sentence of the “Impact on notification” subsection of the Results incorrectly references Fig 2A. This sentence should reference Fig 2. The correct sentence and reference are: The notification rate of new bacteriologically confirmed TB was 368/100,000/yr population before the intervention compared to 620/100,000/yr population after the intervention. (Fig 2 and Table 3)

The last sentence of the “Empirical TB treatment” subsection of the Results incorrectly references Fig 3. This sentence should reference Table 2. The correct sentence and reference are: When restricted to those with Xpert negative results, the median time to starting TB treatment was 12.5 days (IQR 5–32) whilst for those tested with FM it was 7 days (IQR 4–25) (p<0.0001) (Table 2).
